# Evidence for Divergent Selection on Immune Genes between the African Malaria Vectors, *Anopheles coluzzii* and *A. gambiae*

**DOI:** 10.3390/insects11120893

**Published:** 2020-12-18

**Authors:** Yoosook Lee, Lattha Souvannaseng, Travis C. Collier, Bradley J. Main, Laura C. Norris, Abdarahamane Fofana, Sekou F. Traoré, Anthony J. Cornel, Shirley Luckhart, Gregory C. Lanzaro

**Affiliations:** 1 Vector Genetics Laboratory, University of California, Davis, CA 95616, USA; yoosook.lee@ufl.edu (Y.L.); tccollier@ucdavis.edu (T.C.C.); bmain@ucdavis.edu (B.J.M.); laura.norris@gatesfoundation.org (L.C.N.); ajcornel@ucanr.edu (A.J.C.); 2 Department of Pathology, Microbiology and Immunology, School of Veterinary Medicine, University of California, Davis, CA 95616, USA; 3 Florida Medical Entomology Laboratory, University of Florida, Vero Beach, FL 32962, USA; 4 Department of Medical Microbiology and Immunology, University of California, Davis, CA 95616, USA; lsouvannaseng@ucdavis.edu; 5 Malaria Research and Training Center, School of Medicine, University of Bamako, Bamako B.P.2528, Mali; afofana@icermali.org (A.F.); cheick@icermali.org (S.F.T.); 6 Department of Entomology and Nematology, University of California, Davis, CA 95616, USA; 7 Department of Entomology, Plant Pathology and Nematology, University of Idaho, Moscow, ID 83844, USA; sluckhart@uidaho.edu; 8 Department of Biological Sciences, University of Idaho, Moscow, ID 83844, USA

**Keywords:** immune genes, ecological divergence, *Anopheles coluzzii*

## Abstract

**Simple Summary:**

A comparison of the genomes of the African malaria vectors, *Anopheles gambiae* and *A. coluzzii,* revealed that immune genes are highly diverged. Although these two species frequently co-occur within a single site, they occur in distinct larval habitats. Our results taken in the context of known differences in the larval habitats occupied by these taxa support the hypothesis that observed genetic divergence may be driven by immune response to microbial agents specific to these habitats. Strict within species mating may have subsequently evolved in part to maintain immunocompetence which might be compromised by dysregulation of immune pathways in hybrids. We conclude that the evolution of immune gene divergence among this important group of species may serve as a useful model to explore ecological speciation in general.

**Abstract:**

During their life cycles, microbes infecting mosquitoes encounter components of the mosquito anti-microbial innate immune defenses. Many of these immune responses also mediate susceptibility to malaria parasite infection. In West Africa, the primary malaria vectors are *Anopheles coluzzii* and *A. gambiae* sensu stricto, which is subdivided into the Bamako and Savanna sub-taxa. Here, we performed whole genome comparisons of the three taxa as well as genotyping of 333 putatively functional SNPs located in 58 immune signaling genes. Genome data support significantly higher differentiation in immune genes compared with a randomly selected set of non-immune genes among the three taxa (permutation test *p* < 0.001). Among the 58 genes studied, the majority had one or more segregating mutations (72.9%) that were significantly diverged among the three taxa. Genes detected to be under selection include *MAP2K4* and *Raf*. Despite the genome-wide distribution of immune genes, a high level of linkage disequilibrium (r^2^ > 0.8) was detected in over 27% of SNP pairs. We discuss the potential role of immune gene divergence as adaptations to the different larval habitats associated with *A. gambiae* taxa and as a potential force driving ecological speciation in this group of mosquitoes.

## 1. Introduction

Virulence genes in pathogens and immune genes in host/vector species are thought to be co-evolving in an arms race [1,2], resulting in rapid evolution of immune genes, as observed across a broad group of organisms, including primates [3], *Drosophila* species [4,5], and mosquitoes [6]. Differences in selection pressure imposed by distinct microbial environments is likely a major driver of immunological divergence [7].

The immunogenetics of *Anopheles* mosquitoes has been intensively studied, primarily as a means of understanding their role in the transmission of human malaria parasites. Extensive variation in susceptibility to *Plasmodium* infection has been observed among populations of the principal African mosquito vectors [8,9,10]. However, it is likely that any direct selection for *Plasmodium* resistance in these populations is weak due to the generally low infection rate [11,12], variety of different immune signaling pathways involved in resistance [13,14,15,16], and the fact that its innate immune system is co-evolving with many other microbial challenges present in the environment [17]. *Anopheles* mosquitoes experience different immune challenges over the course of development (e.g., larval density, larval nutrition [18,19], and during adult blood feeding [20,21]). It is likely that immune challenges in the larval habitat would be the most important driver of selection [22].

West African populations of *A. gambiae* sensu lato are highly structured and have served as models for the study of speciation with gene flow [23,24,25]. In this paper, we focus on the Bamako and Savanna sub-taxa (known as chromosomal forms) of *A. gambiae* sensu stricto and *A. coluzzii,* formerly referred to as the Mopti form of *A. gambiae* but elevated to species status in 2013 [26]. These three are found in sympatry in Mali [27,28], are largely reproductively isolated [29], and are morphologically indistinguishable so that molecular and/or cytogenetic techniques are required to distinguish them [30,31]. Genomic divergence between *A. coluzzii* and the Bamako and Savanna forms *A. gambiae* is most conspicuous in the centromeric regions in each of their three chromosomes (X, 2, and 3). The metaphor “islands of speciation” has been coined to refer to these genomic regions [24,32]. Although adults of the three taxa are sympatric, they differ with respect to the aquatic environments inhabited by their larvae [33,34,35]. These habitats likely contain distinct microbial communities that drive divergence in immune related genes and, since many of these same genes are related to immunity against *Plasmodium*, this may explain differences in susceptibility to the malaria parasite [6,21,36].

In this study, we set out to test the hypothesis that immune genes show a high degree of divergence. We evaluated a suite of 58 immune genes and compared these with comparable groups of non-immune related genes. In addition to underlying variation in *Plasmodium* susceptibility, immune gene divergence would support the hypothesis that immune challenges are an important selective force driving ecological speciation in this group of mosquitoes [35,37].

We determined genotypes for 333 segregating single nucleotide polymorphisms (SNPs) for each of 471 mosquitoes from the village of Kela, Mali, a site where the Bamako and Savanna forms of *A. gambiae* and *A. coluzzii* occur in sympatry. This panel of SNPs contained either non-synonymous substitutions or resulted in a change in codon frequency with the potential to cause functional variation in the 58 immune genes in which they occur [38,39,40]. The selected immune genes are all located outside the speciation islands; therefore, there is no a priori expectation that they should show elevated divergence among taxa. However, SNPs were observed in all 58 genes tested, and allelic variation was significantly associated with taxa. Additionally, we estimated between-taxon divergence of SNPs in an expansive set of 231 known immune genes using whole-genome sequencing of 12 Bamako, 11 Savanna, and 13 *A. coluzzii* collected from Kela. This allowed us to compare levels of divergence between immune and non-immune genes. Based on these data, we discuss the potential implications of divergence in innate immune genes for maintaining divergence among taxa within the *A. gambiae* complex and as a potential mechanism for speciation in this group.

## 2. Materials and Methods

### 2.1. Sample Collection

Adult female *A. gambiae* s.l. were collected indoors using mouth aspirators between 2009 and 2012 from within a 70 km radius around the village of Kela, Mali (11.89 N, 8.45 W). Collections were made during the rainy season (August–September). Ovaries were removed from semi-gravid females and stored in Carnoy’s solution (1:3 acetic acid:ethanol) for cytogenetic analysis of polytene chromosomes [41]. Abdominal tissues were stored in 70% ethanol for subsequent DNA extraction. Genomic DNAs were extracted using DNeasy^®^ (Qiagen, Valencia, CA, USA).

### 2.2. Species Identification

Members of the *A. gambiae* s.l. species complex (*A. coluzzii* and *A. gambiae*) were distinguished from other *Anopheles* species using an established PCR assay [42]. Specimens of *A. coluzzii* were distinguished from *A. gambiae* s.s. based on an established PCR assay [30,43].

*A. gambiae* is further divided into sub-specific taxa known as the Bamako and Savanna chromosomal forms [27]. These were are identified by karyotyping five paracentric inversions on the right arm of chromosome 2 (2R*j*, 2R*b*, 2R*c*, 2R*d*, and 2R*u*) following standard classification guidelines [27,28]. The polytene chromosomes used for these analyses were extracted from ovarian nurse cells using standard cytogenetic methods [41]. Chromosome banding patterns were examined using an Olympus BX-50 phase contrast microscope (Olympus America, San Jose, CA, USA) and scored relative to the polytene chromosome map for the *A. gambiae* complex [44].

### 2.3. Immune-Related Genes in A. gambiae s.l.

Immune signaling pathways associated with resistance to microbial infection in *Anopheles* mosquitoes involve proteins with functional domains that are highly conserved across metazoans and include the nuclear factor (NF)-κB-dependent Toll and immune deficiency (IMD) pathways [45,46], signaling through Janus kinase/signal transducers and activators of transcription (JAK-STAT) [47], and the mitogen-activated protein kinase (MAPK)-dependent signaling pathways [16,48,49,50]. Signaling from the tumor necrosis factor (TNF) receptor can also modulate these pathways [51,52]. Other signaling pathways involved in growth, metabolism, and development also regulate innate immunity and responses to infection. These include the insulin/insulin-like growth factor signaling (IIS) cascade [14,16,53,54,55] and transforming growth factor (TGF)-beta signaling [56,57,58].

Despite the high degree of conservation of functional domains in these immune signaling pathway proteins, there is variation in susceptibility phenotypes within vector species which can be explained, in part, by genetic variation [8,36,59]. It has been proposed that the ancestral *A. gambiae* phenotype is resistant to *Plasmodium* infection and that a deficiency in immunity is responsible for susceptibility [8]. Studies of Neotropical *Anopheles* species also support this hypothesis [60].

### 2.4. SNP Discovery

A total of 48 individual mosquitoes including 32 *A. gambiae* and 16 *A. coluzzii* were selected for SNP discovery and confirmation. The conserved domains (e.g., catalytic, protein interaction) of a subset of 58 immune-related genes were sequenced. *ChromasLite* ver. 2.01 Technelysium Pty Ltd, South Brisbane, Australia) was used to view chromatograms and convert chromatograms to text sequences. *Geneious* software (Geneious Biologics, West Auckland, New Zealand) was used for sequence alignment. Details are provided in File S1.

### 2.5. SNP Genotyping

From the total of 1947 SNPs discovered, 333 were potentially functional (non-synonymous substitutions or synonymous changes with a 2-fold difference in the codon usage rates, therefore likely to affect folding [38,61]). These were selected for multiplex iPLEX SNP genotyping (Agena Biosciences, San Diego, CA, USA). A total of 254 *A. gambiae s.s.* (167 Bamako form, 87 Savanna form) and 194 *A. coluzzii*, were selected for SNP genotyping. SNP genotyping was conducted at the Veterinary Genetics Laboratory at UC Davis.

### 2.6. Genotype Data Analysis

The significance of the association between genotype and chromosomal form was determined using chi-square tests adjusting for multiple comparisons. Effect size and power calculations were conducted using the *pwr* package in R statistics software [62]. Heterozygosity and F_ST_ were calculated using Arlequin version 3.5 [63]. Loci under selection were detected using the method described by Excoffier et al. [64] as implemented in Arlequin version 3.5 [63]. This method employees a hierarchical island model, in which demes exchange more migrants within than between groups, to generate the joint distribution of genetic diversity within and between populations and greatly reducing false positive loci [64]. Linkage disequilibrium value (r^2^) was calculated using a maximum likelihood method implemented in the EMLD program [65]. Cytoscape [66] was used to draw a gene network.

### 2.7. Whole Genome Sequencing and Data Analysis

We performed whole-genome sequencing on 23 *A. gambiae* s.s. individuals (12 Bamako and 11 Savanna) and 12 *A. coluzzii* individuals all collected from Kela, Mali. We followed the protocol described in Norris et al. [67] for genomic DNA library construction. Genomic DNA libraries were sequenced by the QB3 Vincent J Coates Genomics Sequencing Laboratory at UC Berkeley on the Illumina HiSeq2500 platform with paired-end 100 bp reads. Reads were aligned to the *A. gambiae* reference genome (AgamP3 [68]) with the BWA-MEM aligner [69]. Freebayes v9.9.2-46 [70] was used for SNP identification employing standard filters. F_ST_ values were calculated using the Weir and Cockerham estimator implemented in VCFtools 0.1.12b [71]. Bootstrap *p*-values [72,73] were generated by comparing the F_ST_ value of the set of immune genes to values computed for a random sample of 231 genes (out of 12,519 total genes) repeated 1000 times. Details are provided in File S1.

### 2.8. Data Accessibility

Genome sequencing data have been deposited in the NCBI Sequence Read Archive (SRP062875). Other data associated with this study are available in supporting information at: https://popi.ucdavis.edu/misc/insects/Lee_et_al_2020/.

## 3. Results

Mean heterozygosities for the 333 immune gene SNPs were similar (H = 0.14) in all three taxa with standard deviations of 0.160–0.168, although the number of invariant SNPs was slightly lower in *A. coluzzii* (*n* = 56) than in the Bamako (*n* = 76) or Savanna (*n* = 76) *A. gambiae* populations. Of the 333 SNPs sampled, 100 in 43 genes showed significant associations with taxa (Table 1).

F_ST_ based on SNPs was highest between *A. coluzzii* and Bamako (=0.106) and lowest between the Bamako and Savanna populations (=0.049). Both genome sequence and SNP genotype data consistently indicate that the sub-specific taxa Savanna and Bamako (sub-taxa within *A. gambiae*) are least diverged.

**Figure 1:**
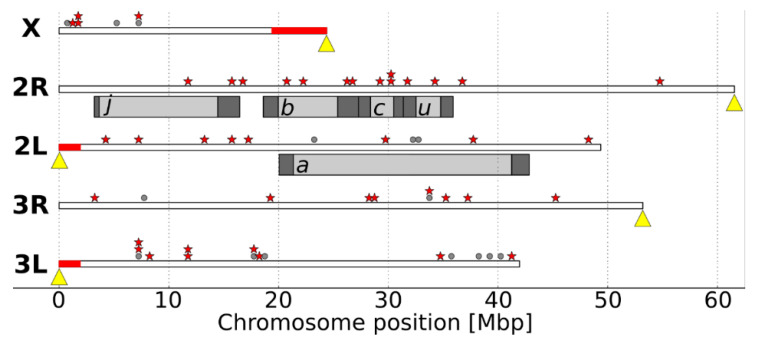
Genome locations of immune genes selected for SNP genotyping depicted by chromosome. Centromere location is marked with a yellow triangle. Red bars indicate the known “speciation islands” of *A. gambiae*. Red stars mark locations of genes that are significantly diverged. Gray circles mark locations of genes that are not significantly differentiated. Gray bars indicate the location of paracentric chromosome inversions, regions containing inversion breakpoints in dark gray.

Paracentric chromosome inversions on chromosome 2R likely contribute to immune gene divergence since 13 of the 14 2R genes studied are located near (<1 Mbp) or within inversion break points (*j*, *b*, *c*, and *u*, indicated in Figure 1).

This significantly exceeds the average number of immune genes expected in these regions if their distributions were random (8.6, +/−1.59, permutation test *p* < 0.05). It is not clear whether immune genes migrate to inversions, similar to genes migrating from the X to autosomes [74,75], or if inversions were selected upon in these regions. We used a hierarchical island model [64] to identify loci under selection and to account for population structure. This model is expected to generate a lower number of markers under selection by removing false positive loci, as compared with non-hierarchical models that are strictly based on F_ST_ values [64]. Although Raf-784 and Raf-851 are only 67 bp apart, we present the genotype distribution of both SNPs because the most common haplotype in each taxon is different. For instance, GC (a concatenation of Raf-784 and Raf-851) is the most common *Raf* haplotype in *A. coluzzii*, the AC haplotype is most common in Bamako, and AA is most common in Savanna). This pattern of taxon-specific divergence in immune signaling genes supports restricted gene flow between taxa that may be influenced by adaptation to different ecological niches [76]. Furthermore, these data indicate that potentially functional divergence is not restricted to pericentric regions [77]. In support of selection driven divergence, three SNPs, MAP2K4-164, Raf-784, and Raf-851, were identified as loci under selection (based on a hierarchical island model [64] *p* < 4.2 × 10^−5^; Table 2).

**Figure 2:**
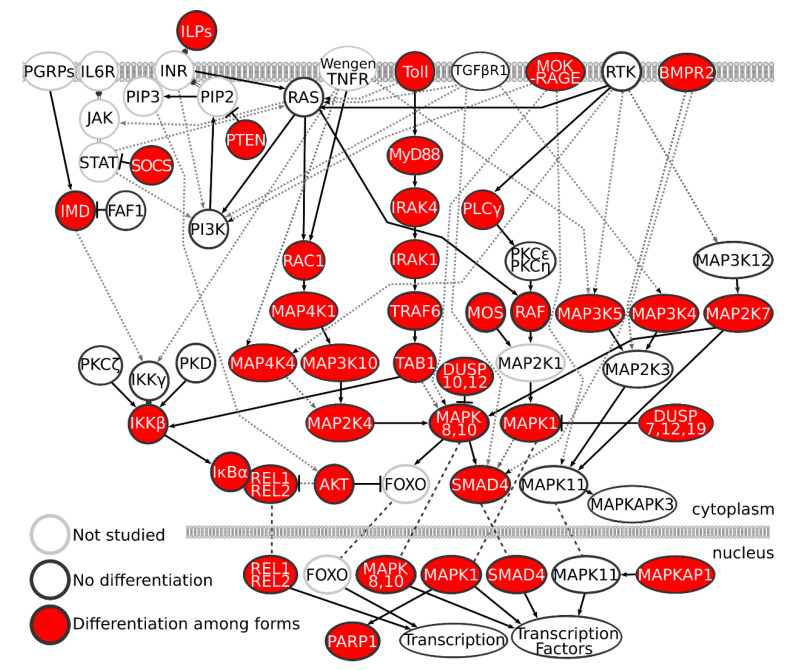
A diagram of immune signaling proteins encoded by genes used for this population-scale SNP genotyping study. Genes that are significantly diverged among the three taxa are in red-filled circles. Black outlined, open circles represent genes with no significant differentiation. Genes in gray outlined, open circles are represented in the pathways but were not included in genotyping. Solid lines indicate direct interactions. Dashed lines indicate indirect activation.

Relatively low linkage disequilibrium (LD) was found among the DUSP19, ILP2, MAPK10, MOK-RAGE, RAS, REL1, TAB1, and Toll5A genes (r^2^ < 0.27 in all pairwise LD), which show varying degrees of pathway networking (Figure 2).

All three taxa have relatively large proportions of SNP pairs (>27%) that are under what we consider high LD (r^2^ > 0.8; Table 3). We also found high LD (mean r^2^ = 0.56) between SNPs with no obvious signaling pathway relationship (Figure 2).

**Figure 3:**
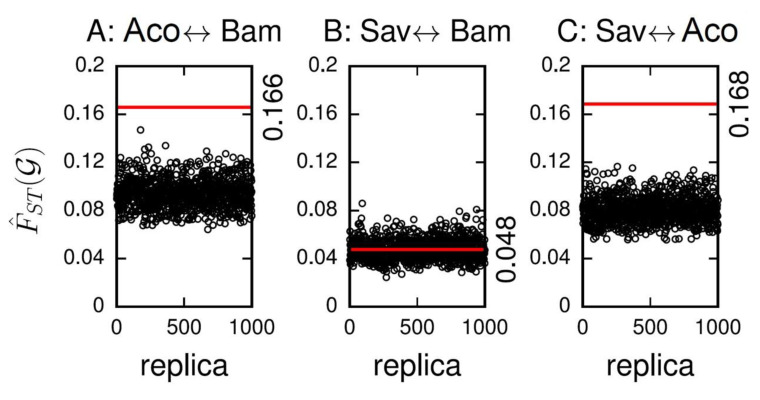
Bootstrap comparison of FST values for immune vs. non-immune genes. The FST value for the set of 231 immune genes (red line; value noted on the right side of each panel) compared with FST values from 1000 randomly selected equal sized sets of non-immune genes (black open circles). Comparisons include (**A**) *A. coluzzii* and Bamako, (**B**) Savanna and Bamako, and (**C**) Savanna and *A. coluzzii.* Aco = *A. coluzzii;* Bam = *A. gambiae,* Bamako form, and Sav = *A. gambiae*, Savanna form.

The level of divergence of SNPs within a comprehensive set of 231 known immune genes was significantly higher (bootstrap *p* < 0.001) than non-immune genes in comparisons between *A. coluzzii* and both *A. gambiae* taxa (Figure 3A,C). Although the overall F_ST_ in the 231 immune genes between Bamako and Savanna forms was non-zero (F_ST_ = 0.037), it was not significantly higher than non-immune related genes (*p* = 0.435; Figure 3B).

The F_ST_ of immune genes was highest between *A. coluzzii* and Savanna (=0.134) and lowest between Bamako and Savanna (=0.037). There were also significantly more (bootstrap *p* = 0.005) SNPs detected within the set of immune genes (=17,712) than a random set of genes (mean ± SEM = 14,408.9 ± 38.1). Genes within the speciation islands were excluded from both the immune and non-immune gene lists, and the same criteria for selecting potentially functional SNPs (non-synonymous or synonymous with a 2-fold difference in usage rate between codons) were applied uniformly.

## 4. Discussion

Divergence between *A. coluzzii* and *A. gambiae* (Savanna + Bamako) has been described as largely restricted to several small, pericentromeric genomic islands of speciation, one on each of the three chromosomes [24,32]. Collectively, these speciation islands represent 3% of the genome, with the remainder of the genome lacking obvious regions of differentiation, presumably due to ongoing gene flow among taxa. Since the immune SNPs analyzed herein lie outside these speciation islands, there is no a priori reason to think they would be highly diverged by, for example, hitchhiking. Additionally, stabilizing selection on potentially functional SNPs would be expected to reduce divergence due to drift. Despite these assumptions, we observed that immune SNPs were significantly associated with taxa. However, fixed mutations were rare, supporting the hypothesis that gene flow is occurring (Supplementary Materials Table S1). Furthermore, most of the immune signaling genes on chromosome 2R are in or near inversions, locations predicted to increase the level of divergence. Based on these observations, we propose that immune gene variation could be driving and maintaining divergence among the three *A. gambiae* taxa within (Table S2).

SNPs in the immune genes analyzed here occur in encoded conserved domains and introduce changes in the amino acid composition in their products (non-synonymous SNP) or were predicted to alter the rate of protein translation and/or folding (synonymous SNP with greater than 2-fold codon frequency change) [38,39,40]. Thus, these mutations could introduce functional changes in the innate immune system that alter survival from basic immune challenges and, coincidentally, susceptibility to *P. falciparum* infection. Susceptibility phenotypes resulting from immune gene SNPs are likely to be influenced by different genetic backgrounds. Furthermore, immune signaling protein variants can perturb multiple signaling networks that impact different cellular processes, thereby leading to outcomes that have indirect rather than direct effects on protein variation [78]. Direct versus indirect effects must be carefully considered when determining if and how naturally occurring nucleotide variants may be causative (as opposed to incidental) given the significant divergence between taxa (Figure 3) and high LD between immune genes (Table 3).

While SNPs that are functionally associated with *P. falciparum* infection have obvious implications for public health, we argue that they could also play a significant role in speciation in this group of mosquitoes. SNPs in immune signaling genes that functionally alter larval responses to environmental pathogens could explain observed larval habitat segregation among *A. gambiae* taxa [34,79,80]. This segregation provides the foundation for an ecologically-based chromosomal speciation hypothesis, which suggests that differing ecological characteristics are driving divergence and adaptation among *A. gambiae* taxa [27,35,81]. A study in Burkina Faso supports this hypothesis by showing that *A. coluzzii* is more often encountered in rice fields, whereas the Savanna taxon (*A. gambiae*) is more often found in small, rainfall-dependent bodies of water [33,79]. The larval habitats of *A. gambiae* s.l., therefore, may harbor diverse microbial communities and the taxa may differ in their tolerance of each. Hybrids between taxa are surprisingly rare in most sympatric sites, and this has been largely attributed to prezygotic isolation [82,83,84]. However, we suggest that mate choice may have evolved in part to maintain a specific immunocompetence by avoiding dysregulation of immune pathways in hybrids [85,86]. In addition, subpar immune function in hybrids may lead to poor survival under natural conditions [32,87].

## 5. Conclusions

Genome comparison of African malaria vectors, *Anopheles gambiae* and *A. coluzzii*, revealed that immune genes are highly diverged. Although sympatric, the two species occur in distinct larval habitats. Our results suggest the hypothesis that divergence is driven by an immune response to microbes specific to the different larval aquatic habitats occupied by these species. Assortative mating may have subsequently evolved in part to maintain immunocompetence, which might be compromised by dysregulation of immune pathways in hybrids. We conclude that the evolution of immune gene divergence among this important group of species may play a critical role in malaria transmission and represent a useful model to explore ecological speciation in general.

## Figures and Tables

**Table 1:** The number of immune genes containing a SNP that differ between at least one pair of taxa.

Chromosome	Diverged Genes	Total Genes Genotyped	% Divergence
X	4	7	57.1%
2L	8	11	72.7%
2R	14	14	100%
3L	9	16	56.3%
3R	8	10	80.0%
Total	43	58	72.9%

**Table 2:** Distribution of SNP genotypes among potentially functional SNPs. Results support the hypothesis that these are under significant selection among taxa (α < 0.05). Numbers indicated number of individuals observed per genotype.

Chromosome	SNP	Genotype	Ac	Bam	Sav
2R	MAP2K4-164	C/C	164	13	58
		C/T	1	74	27
		T/T	0	87	1
2L	Raf-784	A/A	22	128	82
		A/G	75	3	0
		G/G	55	0	0
2L	Raf-851	A/A	12	2	69
		A/C	78	20	2
		C/C	60	106	3

Ac stands for *A. coluzzii*, Bam for Bamako sub-taxon of *A. gambiae* and Sav for Savanna sub-taxon of *A. gambiae*.

**Table 3:** Proportions of potentially functional SNP pairs where linkage disequilibrium (LD; r^2^) exceeds 0.5, 0.8, and 0.95 thresholds based on population-scale genotype data.

Forms	r^2^ > 0.5	r^2^ > 0.8	r^2^ > 0.95
**LD between genes**			
*A. gambiae* (Bamako)	32.1%	28.6%	25.2%
*A. coluzzii*	30.6%	27.4%	23.8%
*A. gambiae* (Savanna)	32.7%	29.7%	26.0%
**LD within a gene**			
*A. gambiae* (Bamako)	35.4%	30.9%	27.1%
*A. coluzzii*	34.3%	28.4%	25.9%
*A. gambiae* (Savanna)	34.8%	31.3%	26.7%

## Supplementary Materials

The following are available online at https://www.mdpi.com/2075-4450/11/12/893/s1, File S1: Materials and Methods (https://popi.ucdavis.edu/misc/insects/Lee_et_al_2020/), Table S1: Genotype data, Table S2: List of immune and other genes used in divergence (FST) estimation based on whole-genome sequencing.

## References

[1] Wegner K.M., Kalbe M., Milinski M., Reusch T.B. (2008). Mortality selection during the 2003 European heat wave in three-spined sticklebacks: Effects of parasites and MHC genotype. BMC Evol. Biol..

[2] Lazzaro B.P., Little T.J. (2009). Immunity in a variable world. Philos. Trans. R. Soc. Lond. Ser. B Biol. Sci..

[3] Nielsen R., Bustamante C., Clark A.G., Glanowski S., Sackton T.B., Hubisz M.J., Fledel-Alon A., Tanenbaum D.M., Civello D., White T.J. (2005). A scan for positively selected genes in the genomes of humans and chimpanzees. PLoS Biol..

[4] Schlenke T.A., Begun D.J. (2003). Natural selection drives *Drosophila* immune system evolution. Genetics.

[5] Obbard D.J., Welch J.J., Kim K.W., Jiggins F.M. (2009). Quantifying adaptive evolution in the *Drosophila* immune system. PLoS Genet..

[6] Crawford J.E., Bischoff E., Garnier T., Gneme A., Eiglmeier K., Holm I., Riehle M.M., Guelbeogo W.M., Sagnon N., Lazzaro B.P. (2012). Evidence for population-specific positive selection on immune genes of *Anopheles gambiae*. G3.

[7] Sackton T.B., Lazzaro B.P., Schlenke T.A., Evans J.D., Hultmark D., Clark A.G. (2007). Dynamic evolution of the innate immune system in *Drosophila*. Nat. Genet..

[8] Riehle M.M., Markianos K., Niare O., Xu J., Li J., Toure A.M., Podiougou B., Oduol F., Diawara S., Diallo M. (2006). Natural malaria infection in *Anopheles gambiae* is regulated by a single genomic control region. Science.

[9] Fryxell R.T., Nieman C.C., Fofana A., Lee Y., Traore S.F., Cornel A.J., Luckhart S., Lanzaro G.C. (2012). Differential *Plasmodium falciparum* infection of *Anopheles gambiae* s.s. molecular and chromosomal forms in Mali. Malar. J..

[10] Ndiath M.O., Cohuet A., Gaye A., Konate L., Mazenot C., Faye O., Boudin C., Sokhna C., Trape J.F. (2011). Comparative susceptibility to *Plasmodium falciparum* of the molecular forms M and S of *Anopheles gambiae* and *Anopheles arabiensis*. Malar. J..

[11] Beier J.C., Killeen G.F., Githure J.I. (1999). Short report: Entomologic inoculation rates and *Plasmodium falciparum* malaria prevalence in Africa. Am. J. Trop. Med. Hyg..

[12] Dia I., Ba H., Mohamed S.A., Diallo D., Lo B., Diallo M. (2009). Distribution, host preference and infection rates of malaria vectors in Mauritania. Parasites Vectors.

[13] Clayton A.M., Dong Y., Dimopoulos G. (2014). The *Anopheles* innate immune system in the defense against malaria infection. J. Innate Immun..

[14] Surachetpong W., Pakpour N., Cheung K.W., Luckhart S. (2011). Reactive oxygen species-dependent cell signaling regulates the mosquito immune response to *Plasmodium falciparum*. Antioxid. Redox Signal..

[15] Corby-Harris V., Drexler A., Watkins de Jong L., Antonova Y., Pakpour N., Ziegler R., Ramberg F., Lewis E.E., Brown J.M., Luckhart S. (2010). Activation of Akt signaling reduces the prevalence and intensity of malaria parasite infection and lifespan in *Anopheles stephensi* mosquitoes. PLoS Pathog.

[16] Souvannaseng L., Hun L.V., Baker H., Klyver J.M., Wang B., Pakpour N., Bridgewater J.M., Napoli E., Giulivi C., Riehle M.A. (2018). Inhibition of JNK signaling in the Asian malaria vector *Anopheles stephensi* extends mosquito longevity and improves resistance to *Plasmodium falciparum* infection. PLoS Pathog.

[17] Weiss B., Aksoy S. (2011). Microbiome influences on insect host vector competence. Trends Parasitol..

[18] Telang A., Qayum A.A., Parker A., Sacchetta B.R., Byrnes G.R. (2012). Larval nutritional stress affects vector immune traits in adult yellow fever mosquito *Aedes aegypti* (*Stegomyia aegypti*). Med. Vet. Entomol..

[19] Muturi E.J., Blackshear M., Montgomery A. (2012). Temperature and density-dependent effects of larval environment on *Aedes aegypti* competence for an alphavirus. J. Vector Ecol..

[20] Hillyer J.F., Estevez-Lao T.Y. (2010). Nitric oxide is an essential component of the hemocyte-mediated mosquito immune response against bacteria. Dev. Comp. Immunol..

[21] Okech B.A., Gouagna L.C., Yan G., Githure J.I., Beier J.C. (2007). Larval habitats of *Anopheles gambiae* s.s. (Diptera: Culicidae) influences vector competence to *Plasmodium falciparum* parasites. Malar. J..

[22] Rottschaefer S.M., Crawford J.E., Riehle M.M., Guelbeogo W.M., Gneme A., Sagnon N., Vernick K.D., Lazzaro B.P. (2015). Population Genetics of *Anopheles coluzzii* Immune Pathways and Genes. G3 Genes Genom. Genet..

[23] della Torre A., Costantini C., Besansky N.J., Caccone A., Petrarca V., Powell J.R., Coluzzi M. (2002). Speciation within *Anopheles gambiae*—The glass is half full. Science.

[24] Turner T.L., Hahn M.W., Nuzhdin S.V. (2005). Genomic islands of speciation in *Anopheles gambiae*. PLoS Biol..

[25] Lanzaro G.C., Lee Y., Manguin S. (2013). Speciation in *Anopheles gambiae*—The Distribution of Genetic Polymorphism and Patterns of Reproductive Isolation Among Natural Populations. Anopheles Mosquitoes—New Insights into Malaria Vector.

[26] Coetzee M., Hunt R.H., Wilkerson R., Della Torre A., Coulibaly M.B., Besansky N.J. (2013). *Anopheles coluzzii* and *Anopheles amharicus*, new members of the *Anopheles gambiae* complex. Zootaxa.

[27] Touré Y.T., Petrarca V., Traoré S.F., Coulibaly A., Maiga H.M., Sankare O., Sow M., Di Deco M.A., Coluzzi M. (1998). The distribution and inversion polymorphism of chromosomally recognized taxa of the *Anopheles gambiae* complex in Mali, West Africa. Parassitologia.

[28] Lee Y., Collier T.C., Sanford M.R., Marsden C.D., Fofana A., Cornel A.J., Lanzaro G.C. (2013). Chromosome inversions, genomic differentiation and speciation in the African malaria mosquito *Anopheles gambiae*. PLoS ONE.

[29] Taylor C., Touré Y.T., Carnahan J., Norris D.E., Dolo G., Traoré S.F., Edillo F.E., Lanzaro G.C. (2001). Gene flow among populations of the malaria vector, *Anopheles gambiae*, in Mali, West Africa. Genetics.

[30] Favia G., della Torre A., Bagayoko M., Lanfrancotti A., Sagnon N., Toure Y.T., Coluzzi M. (1997). Molecular identification of sympatric chromosomal forms of *Anopheles gambiae* and further evidence of their reproductive isolation. Insect Mol. Biol..

[31] Fanello C., Santolamazza F., della Torre A. (2002). Simultaneous identification of species and molecular forms of the *Anopheles gambiae* complex by PCR-RFLP. Med. Vet. Entomol..

[32] White B.J., Cheng C., Simard F., Costantini C., Besansky N.J. (2010). Genetic association of physically unlinked islands of genomic divergence in incipient species of *Anopheles gambiae*. Mol. Ecol..

[33] Gimonneau G., Pombi M., Choisy M., Morand S., Dabire R.K., Simard F. (2012). Larval habitat segregation between the molecular forms of the mosquito *Anopheles gambiae* in a rice field area of Burkina Faso, West Africa. Med. Vet. Entomol..

[34] Sanford M.R., Ramsay S., Cornel A.J., Marsden C.D., Norris L.C., Patchoke S., Fondjo E., Lanzaro G.C., Lee Y. (2013). A preliminary investigation of the relationship between water quality and *Anopheles gambiae* larval habitats in Western Cameroon. Malar. J..

[35] Manoukis N.C., Powell J.R., Toure M.B., Sacko A., Edillo F.E., Coulibaly M.B., Traore S.F., Taylor C.E., Besansky N.J. (2008). A test of the chromosomal theory of ecotypic speciation in *Anopheles gambiae*. Proc. Natl. Acad. Sci. USA.

[36] Riehle M.M., Guelbeogo W.M., Gneme A., Eiglmeier K., Holm I., Bischoff E., Garnier T., Snyder G.M., Li X., Markianos K. (2011). A cryptic subgroup of *Anopheles gambiae* is highly susceptible to human malaria parasites. Science.

[37] Coluzzi M., Petrarca V., Di Deco M. (1985). Chromosomal inversion intergradation and incipient speciation in *Anopheles gambiae*. Boll. Zool..

[38] Kimchi-Sarfaty C., Oh J.M., Kim I.W., Sauna Z.E., Calcagno A.M., Ambudkar S.V., Gottesman M.M. (2007). A “silent“ polymorphism in the MDR1 gene changes substrate specificity. Science.

[39] Purvis I.J., Bettany A.J., Santiago T.C., Coggins J.R., Duncan K., Eason R., Brown A.J. (1987). The efficiency of folding of some proteins is increased by controlled rates of translation in vivo. A hypothesis. J. Mol. Biol..

[40] Brenton A.A., Souvannaseng L., Cheung K., Anishchenko M., Brault A.C., Luckhart S. (2014). Engineered single nucleotide polymorphisms in the mosquito MEK docking site alter *Plasmodium berghei* development in *Anopheles gambiae*. Parasites Vectors.

[41] Hunt R.H. (1973). A cytological technique for the study of *Anopheles gambiae* complex. Parassitologia.

[42] Scott J.A., Brogdon W.G., Collins F.H. (1993). Identification of single specimens of the *Anopheles gambiae* complex by the polymerase chain reaction. Am. J. Trop. Med. Hyg..

[43] Favia G., Lanfrancotti A., Spanos L., Siden-Kiamos I., Louis C. (2001). Molecular characterization of ribosomal DNA polymorphisms discriminating among chromosomal forms of *Anopheles gambiae* s.s. Insect Mol. Biol..

[44] Coluzzi M., Sabatini A., della Torre A., Di Deco M.A., Petrarca V. (2002). A polytene chromosome analysis of the *Anopheles gambiae* species complex. Science.

[45] Christophides G.K., Zdobnov E., Barillas-Mury C., Birney E., Blandin S., Blass C., Brey P.T., Collins F.H., Danielli A., Dimopoulos G. (2002). Immunity-related genes and gene families in *Anopheles gambiae*. Science.

[46] Garver L.S., Bahia A.C., Das S., Souza-Neto J.A., Shiao J., Dong Y., Dimopoulos G. (2012). *Anopheles* Imd pathway factors and effectors in infection intensity-dependent anti-*Plasmodium* action. PLoS Pathog.

[47] Barillas-Mury C., Han Y.S., Seeley D., Kafatos F.C. (1999). *Anopheles gambiae* Ag-STAT, a new insect member of the STAT family, is activated in response to bacterial infection. EMBO J..

[48] Wang B., Pakpour N., Napoli E., Drexler A., Glennon E.K., Surachetpong W., Cheung K., Aguirre A., Klyver J.M., Lewis E.E. (2015). *Anopheles stephensi* p38 MAPK signaling regulates innate immunity and bioenergetics during *Plasmodium falciparum* infection. Parasites Vectors.

[49] Surachetpong W., Singh N., Cheung K.W., Luckhart S. (2009). MAPK ERK signaling regulates the TGF-beta1-dependent mosquito response to *Plasmodium falciparum*. PLoS Pathog.

[50] Pietri J.E., Cheung K.W., Luckhart S. (2014). Knockdown of mitogen-activated protein kinase (MAPK) signalling in the midgut of *Anopheles stephensi* mosquitoes using antisense morpholinos. Insect Mol. Biol..

[51] Ferrandon D., Imler J.L., Hetru C., Hoffmann J.A. (2007). The *Drosophila* systemic immune response: Sensing and signalling during bacterial and fungal infections. Nat. Rev. Immunol..

[52] Napetschnig J., Wu H. (2013). Molecular basis of NF-kappaB signaling. Annu. Rev. Biophys..

[53] Lim J., Gowda D.C., Krishnegowda G., Luckhart S. (2005). Induction of nitric oxide synthase in *Anopheles stephensi* by *Plasmodium falciparum*: Mechanism of signaling and the role of parasite glycosylphosphatidylinositols. Infect Immun..

[54] Luckhart S., Giulivi C., Drexler A.L., Antonova-Koch Y., Sakaguchi D., Napoli E., Wong S., Price M.S., Eigenheer R., Phinney B.S. (2013). Sustained activation of Akt elicits mitochondrial dysfunction to block *Plasmodium falciparum* infection in the mosquito host. PLoS Pathog.

[55] Pakpour N., Corby-Harris V., Green G.P., Smithers H.M., Cheung K.W., Riehle M.A., Luckhart S. (2012). Ingested human insulin inhibits the mosquito NF-kappaB-dependent immune response to *Plasmodium falciparum*. Infect Immun..

[56] Crampton A., Luckhart S. (2001). The role of As60A, a TGF-beta homolog, in Anopheles stephensi innate immunity and defense against Plasmodium infection. Infect. Genet. Evol. J. Mol. Epidemiol. Evol. Genet. Infect. Dis..

[57] Price I., Ermentrout B., Zamora R., Wang B., Azhar N., Mi Q., Constantine G., Faeder J.R., Luckhart S., Vodovotz Y. (2013). In vivo, in vitro, and in silico studies suggest a conserved immune module that regulates malaria parasite transmission from mammals to mosquitoes. J. Theor. Biol..

[58] Vodovotz Y., Zamora R., Lieber M.J., Luckhart S. (2004). Cross-talk between nitric oxide and transforming growth factor-beta1 in malaria. Curr. Mol. Med..

[59] Collins F.H., Sakai R.K., Vernick K.D., Paskewitz S., Seeley D.C., Miller L.H., Collins W.E., Campbell C.C., Gwadz R.W. (1986). Genetic selection of a Plasmodium-refractory strain of the malaria vector *Anopheles gambiae*. Science.

[60] Rios-Velasquez C.M., Martins-Campos K.M., Simoes R.C., Izzo T., Dos Santos E.V., Pessoa F.A., Lima J.B., Monteiro W.M., Secundino N.F., Lacerda M.V. (2013). Experimental *Plasmodium vivax* infection of key *Anopheles* species from the Brazilian Amazon. Malar. J..

[61] Komar A.A., Lesnik T., Reiss C. (1999). Synonymous codon substitutions affect ribosome traffic and protein folding during in vitro translation. FEBS Lett..

[62] R Core Team The R Project for Statistical Computing. http://www.r-project.org/.

[63] Excoffier L., Lischer H.E. (2010). Arlequin suite ver 3.5: A new series of programs to perform population genetics analyses under Linux and Windows. Mol. Ecol. Resour..

[64] Excoffier L., Hofer T., Foll M. (2009). Detecting loci under selection in a hierarchically structured population. Heredity.

[65] Huang Q., Shete S., Swartz M., Amos C.I. (2005). Examining the effect of linkage disequilibrium on multipoint linkage analysis. BMC Genet..

[66] Shannon P., Markiel A., Ozier O., Baliga N.S., Wang J.T., Ramage D., Amin N., Schwikowski B., Ideker T. (2003). Cytoscape: A software environment for integrated models of biomolecular interaction networks. Genome Res..

[67] Norris L.C., Main B.J., Lee Y., Collier T.C., Fofana A., Cornel A.J., Lanzaro G.C. (2015). Adaptive introgression in an African malaria mosquito coincident with the increased usage of insecticide-treated bed nets. Proc. Natl. Acad. Sci. USA.

[68] Giraldo-Calderon G.I., Emrich S.J., MacCallum R.M., Maslen G., Dialynas E., Topalis P., Ho N., Gesing S., VectorBase C., Madey G. (2015). VectorBase: An updated bioinformatics resource for invertebrate vectors and other organisms related with human diseases. Nucleic Acids Res..

[69] Li H. (2013). Aligning sequence reads, clone sequences and assembly contigs with BWA-MEM. arXiv.

[70] Li H., Handsaker B., Wysoker A., Fennell T., Ruan J., Homer N., Marth G., Abecasis G., Durbin R., Genome Project Data Processing S. (2009). The Sequence Alignment/Map format and SAMtools. Bioinformatics.

[71] Danecek P., Auton A., Abecasis G., Albers C.A., Banks E., DePristo M.A., Handsaker R.E., Lunter G., Marth G.T., Sherry S.T. (2011). The variant call format and VCFtools. Bioinformatics.

[72] Davison A.C., Hinkley D.V. (1997). Bootstrap Methods and Their Application.

[73] North B.V., Curtis D., Sham P.C. (2002). A note on the calculation of empirical P values from Monte Carlo procedures. Am. J. Hum. Genet..

[74] Betran E., Thornton K., Long M. (2002). Retroposed new genes out of the X in *Drosophila*. Genome Res..

[75] Emerson J.J., Kaessmann H., Betran E., Long M. (2004). Extensive gene traffic on the mammalian X chromosome. Science.

[76] Schmid-Hempel P. (2003). Variation in immune defence as a question of evolutionary ecology. Proc. Biol. Sci. R. Soc..

[77] Lawniczak M.K., Emrich S.J., Holloway A.K., Regier A.P., Olson M., White B., Redmond S., Fulton L., Appelbaum E., Godfrey J. (2010). Widespread divergence between incipient *Anopheles gambiae* species revealed by whole genome sequences. Science.

[78] Karczewski K.J., Dudley J.T., Kukurba K.R., Chen R., Butte A.J., Montgomery S.B., Snyder M. (2013). Systematic functional regulatory assessment of disease-associated variants. Proc. Natl. Acad. Sci. USA.

[79] Diabate A., Dabire R.K., Kim E.H., Dalton R., Millogo N., Baldet T., Simard F., Gimnig J.E., Hawley W.A., Lehmann T. (2005). Larval development of the molecular forms of *Anopheles gambiae* (Diptera: Culicidae) in different habitats: A transplantation experiment. J. Med. Entomol..

[80] League G.P., Estévez-Lao T.Y., Yan Y., Garcia-Lopez V.A., Hillyer J.F. (2017). *Anopheles gambiae* larvae mount stronger immune responses against bacterial infection than adults: Evidence of adaptive decoupling in mosquitoes. Parasites Vectors.

[81] Coluzzi M., Sabatini A., Petrarca V., Di Deco M.A. (1979). Chromosomal differentiation and adaptation to human environments in the *Anopheles gambiae* complex. Trans. R Soc. Trop. Med. Hyg..

[82] Persiani A., Di Deco M.A., Petrangeli G. (1986). Laboratory observation of inversion polymorphisms originating from the crossing of various populations of *Anopheles gambiae* s.s. Ann. Ist. Super Sanita.

[83] Diabate A., Dabire R.K., Millogo N., Lehmann T. (2007). Evaluating the effect of postmating isolation between molecular forms of *Anopheles gambiae* (Diptera: Culicidae). J. Med. Entomol..

[84] Pennetier C., Warren B., Dabire K.R., Russell I.J., Gibson G. (2010). “Singing on the wing” as a mechanism for species recognition in the malarial mosquito *Anopheles gambiae*. Curr. Biol..

[85] Lazzaro B.P., Clark A.G. (2003). Molecular population genetics of inducible antibacterial peptide genes in *Drosophila melanogaster*. Mol. Biol. Evol..

[86] Hill-Burns E.M., Clark A.G. (2010). Functional regulatory divergence of the innate immune system in interspecific *Drosophila* hybrids. Mol. Biol. Evol..

[87] Lee Y., Marsden C.D., Norris L.C., Collier T.C., Main B.J., Fofana A., Cornel A.J., Lanzaro G.C. (2013). Spatiotemporal dynamics of gene flow and hybrid fitness between the M and S forms of the malaria mosquito, *Anopheles gambiae*. Proc. Natl. Acad. Sci. USA.

