# Supplementary material for: Evidence for Divergent Selection on Immune Genes between the African Malaria Vectors, Anopheles coluzzii and A. gambiae
Source: Insects. 2020 Dec 18;11(12):893. doi: 10.3390/insects11120893 (PMC7767042; doi:10.3390/insects11120893)
Supplement: Supplementary file 1 [file insects-11-00893-s001.zip › insects-999272-supplementary/insects-999272-supple-conversion/File S1.docx]

**File S1**

**Materials and Methods**

SNP discovery

A total of 48 individual mosquitoes including 32 *A. gambiae* and 16 *A. coluzzii* were selected for SNP discovery and confirmation. Orthologs of human and fruit fly genes with established roles in innate immunity and anti-pathogen signaling were identified in the *A. gambiae* genome (PEST strain) as previously described ([1](#_ENREF_1)). All genes were confirmed using Basic Local Alignment Search Tool (BLAST) ([2](#_ENREF_2)) and conserved domains of each predicted protein sequence were identified using the Conserved Domain Database (CDD) ([3](#_ENREF_3)). Up to five DNA sequencing primer pairs were designed using Primer3 (<http://frodo.wi.mit.edu/primer3/>). For optimal sequencing results, we limited GC content of each primer to 45-60% and primer melting temperature to 57-63°C.

The conserved domains (e.g., catalytic, protein interaction) of a total of 58 immune-related genes (94 PCR amplimers) were sequenced of the immune-related genes listed in (Table S2). A signaling network diagram for the protein products of these genes is provided in Figure 2.

Each 50 µl PCR contained 0.5 µM of forward and reverse primers, 1X PCR buffer (Applied Biosystems, Carlsbad, California), 1.5 mM MgCl_2_, 200 µM dNTP mix, 1.25 U Ampli*Taq* DNA polymerase (Applied Biosystems, Carlsbad, CA) and 2 µl of DNA template. The thermocycler was programmed to denature for 5 min at 95˚C followed by 35 cycles of 95˚C for 30 sec, 48-54˚C for 30 sec, 72˚C for 30 sec and then a final extension for 5 min at 72˚C. For each amplimer, the reaction was adjusted as necessary by either modifying the PCR mix and/or thermal cycling annealing conditions for optimal amplification. Amplicons were sequenced at the ^UC^DNA Sequencing Facility (College of Biological Sciences, UC Davis) using an ABI 3730 Genetic Analyzer (Applied Biosystems, Carlsbad, California). *ChromasLite* ver. 2.01 was used to view chromatograms and convert chromatograms to text sequences. *Geneious* software was used for sequence alignment.

**Table SI-1.** The immune genes investigated using population-scale genotyping. NS stands for the number of non-synonymous SNPs genotyped for each corresponding gene. S stands for the number of synonymous genotyped. The number of SNPs that showed divergence among chromosomal forms is noted in parentheses. Genes with at least one SNP with significant divergence among chromosomal forms are noted as “Y”.

| idx | chr | gname | Ref. | AGAP0 | domain | Form divergence | NS | S |
| --- | --- | --- | --- | --- | --- | --- | --- | --- |
| 1 | X | PKD | ([4](#_ENREF_4)) | 00040 | MFS |  | 1 | 5 |
| 2 | X | MAP2K3 | ([1](#_ENREF_1)) | 00310 | PKc_like, S_TKc |  | 0 | 2 |
| 3 | X | TRAF6 | ([5](#_ENREF_5)) | 00388 | MATH_TRAF_C | Y | 2 (1) | 4 |
| 4 | X | PKCη | ([4](#_ENREF_4)) | 00418 | PKc_like, S_TKc |  | 0 | 5 |
| 5 | X | MAP3K5 | ([1](#_ENREF_1)) | 00747 | DUF4071 | Y | 0 | 3 (1) |
| 6 | X | Toll5A | ([6](#_ENREF_6)) | 00999 | TIR | Y | 2 (1) | 1 |
| 7 | 2R | MAP2K7 | ([1](#_ENREF_1)) | 01867 | PKc_MKK7, S_TKc | Y | 1 | 4 (3) |
| 8 | 2R | DUSP12 | ([7](#_ENREF_7), [8](#_ENREF_8)) | 02108 | DSPc | Y | 1 | 6 (2) |
| 9 | 2R | AKT | ([9-15](#_ENREF_9)) | 02161 | PH-like super family | Y | 0 | 3 (1) |
| 10 | 2R | MAP3K4 | ([1](#_ENREF_1)) | 02371 | STKc_MEKK4 | Y | 0 | 4 (1) |
| 11 | 2R | MOK-RAGE | ([16](#_ENREF_16)) | 02515 | STKc_MOK | Y | 2 | 7 (3) |
| 12 | 2R | MAP3K10 | ([1](#_ENREF_1)) | 02710 | SH3_MKL, STYKc, PTKc | Y | 0 | 4 (1) |
| 13 | 2R | PKCε | ([4](#_ENREF_4)) | 02748 | STKc_nPKC_epsilon | Y | 1 | 2 (1) |
| 14 | 2R | SMAD4 | ([17-24](#_ENREF_17)) | 02902 | MH1, MH2 | Y | 1 | 4 (2) |
| 15 | 2R | TAB1 | ([17-24](#_ENREF_17)) | 02953 | PP2Cc | Y | 1 | 7 (6) |
| 16 | 2R | IRAK1 | ([5](#_ENREF_5)) | 02966 | Death_Pelle | Y | 2 (1) | 1 (1) |
| 17 | 2R | IRAK4 | ([5](#_ENREF_5)) | 03062 | Death_Tube, DD_superfamily, PKc | Y | 5 (4) | 9 (3) |
| 18 | 2R | PARP1 | ([5](#_ENREF_5)) | 03230 | parp_like | Y | 1 (1) | 8 (6) |
| 19 | 2R | MAP2K4 | ([1](#_ENREF_1)) | 03365 | PKc_like super family, PKc_MKK4 | Y | 0 | 2 (2) |
| 20 | 2R | DUSP10 | ([7](#_ENREF_7), [8](#_ENREF_8)) | 04353 | DSPc | Y | 1 | 5 (2) |
| 21 | 2L | Raf | ([25](#_ENREF_25)) | 04699 | PKc | Y | 1 | 5 (5) |
| 22 | 2L | SOCS44A | ([25](#_ENREF_25)) | 04844 | SH2_SOCS_family | Y | 5 (5) | 2 (2) |
| 23 | 2L | IMD | ([6](#_ENREF_6)) | 04959 | Death | Y | 2 | 4 (2) |
| 24 | 2L | MYD88 | ([5](#_ENREF_5)) | 05252 | TIR | Y | 1 | 3 (2) |
| 25 | 2L | RAC1 | ([4](#_ENREF_4)) | 05445 | RAC1_like | Y | 0 | 5 (1) |
| 26 | 2L | BMPR2 | ([17-24](#_ENREF_17)) | 05567 | PKc, Pkinase | Y | 0 | 3 (2) |
| 27 | 2L | IKKγ | ([5](#_ENREF_5)) | 05898 | STKc_NLK |  | 1 | 5 |
| 28 | 2L | MAP4K4 | ([1](#_ENREF_1)) | 06340 | STKc_myosinIII_like, CNH | Y | 1 | 7 (1) |
| 29 | 2L | MAP3K12 | ([1](#_ENREF_1)) | 06461 | PTKc, TyrKc |  | 1 | 2 |
| 30 | 2L | FAF1 | ([5](#_ENREF_5)) | 06473 | UAS_FAF1 |  | 0 | 4 |
| 31 | 2L | REL2 | ([5](#_ENREF_5)) | 06747 | RHD-n_Relish | Y | 2 (1) | 1 |
| 32 | 2L | MOS | ([26](#_ENREF_26)) | 07598 | PKc | Y | 3 (1) | 1 |
| 33 | 3R | IκBα | ([5](#_ENREF_5)) | 07938 | ANK | Y | 5 (1) | 0 |
| 34 | 3R | TGFBR1 | ([17-24](#_ENREF_17)) | 08247 | PKc, PTKc_VEGFR, Pkinase |  | 1 | 2 |
| 35 | 3R | MAPKAP1 | ([27](#_ENREF_27)) | 08831 | SIN1 | Y | 4 | 12 (1) |
| 36 | 3R | IKKβ | ([5](#_ENREF_5)) | 09166 | S_TKc | Y | 1 | 2 (1) |
| 37 | 3R | MAPK1 | ([1](#_ENREF_1)) | 09207 | STKc_ERK1_2_like | Y | 0 | 1 (1) |
| 38 | 3R | MAPK10 | ([1](#_ENREF_1)) | 09460 | STKc_JNK, PKc_like super family |  | 3 | 2 |
| 39 | 3R | MAPK8 | ([1](#_ENREF_1)) | 09461 | STKc_JNK | Y | 2 | 5 (1) |
| 40 | 3R | REL1 | ([5](#_ENREF_5)) | 09515 | IPT_NFkappaB | Y | 1 | 5 (3) |
| 41 | 3R | PTEN | ([9-15](#_ENREF_9)) | 09628 | CDC14 | Y | 1 | 2 (1) |
| 42 | 3R | DUSP19 | ([7](#_ENREF_7), [8](#_ENREF_8)) | 09903 | DSPc | Y | 3 (1) | 2 |
| 43 | 3L | ILP2 | ([9-15](#_ENREF_9)) | 10600 | Insulin/IGF/Relaxin family, IIGF_like super family | Y | 1 | 3 (1) |
| 44 | 3L | ILP4 | ([9-15](#_ENREF_9)) | 10601 | IIGF_insulin_bombyxin_like | Y | 3 | 3 (1) |
| 45 | 3L | ILP3 | ([9-15](#_ENREF_9)) | 10604 | IIGF_insulin_bombyxin_like |  | 0 | 3 |
| 46 | 3L | Toll5B | ([6](#_ENREF_6)) | 10669 | PLN00113, LRR_RI | Y | 18 (1) | 4 (1) |
| 47 | 3L | MAP4K1 | ([1](#_ENREF_1)) | 10837 | STKc_MAP4K3_like | Y | 1 (1) | 5 (2) |
| 48 | 3L | SOCS36E | ([25](#_ENREF_25)) | 11042 | SH2_SOCS_family | Y | 0 | 6 (2) |
| 49 | 3L | RAS | ([25](#_ENREF_25)) | 11133 | PTZ00314,TIM_phosphphate_binding |  | 0 | 2 |
| 50 | 3L | PLCγ | ([4](#_ENREF_4)) | 11152 | PH_PLC | Y | 1 | 4 (1) |
| 51 | 3L | Toll11 | ([6](#_ENREF_6)) | 11186 | LRR_8, LRR_RI, TIR | Y | 2 (1) | 9 (2) |
| 52 | 3L | Toll10 | ([6](#_ENREF_6)) | 11187 | LRR, LRR_8, LRR_RI |  | 0 | 5 |
| 53 | 3L | MAPKAPK3 | ([28](#_ENREF_28), [29](#_ENREF_29)) | 11890 | STKc_AGC, PKc_like super family | Y | 2 | 4 (1) |
| 54 | 3L | PKCζ | ([4](#_ENREF_4)) | 11993 | PB1_aPKC |  | 0 | 2 |
| 55 | 3L | MAPK11 | ([1](#_ENREF_1)) | 12148 | PKc_like |  | 0 | 1 |
| 56 | 3L | DUSP7 | ([7](#_ENREF_7), [8](#_ENREF_8)) | 12237 | DSP_MapKP, DSPc |  | 0 | 4 |
| 57 | 3L | Toll7 | ([6](#_ENREF_6)) | 12326 | LRR_RI, PLN00113, LRRCT |  | 1 | 10 |
| 58 | 3L | Toll6 | ([6](#_ENREF_6)) | 12387 | PLN00113, LRR_RI, TIR | Y | 1 (1) | 14 |

Whole genome sequencing and data analysis

We performed whole-genome sequencing on 12 Bamako, 12 Mopti and 11 Savanna form mosquitoes collected from Kela, Mali. We followed the protocol described in Norris et al. ([30](#_ENREF_30)) for genomic DNA library construction. Genomic DNA libraries were sequenced by the QB3 Vincent J Coates Genomics Sequencing Laboratory at UC Berkeley on the Illumina HiSeq2500 platform with paired-end 100 bp reads.

Adaptor sequences and poor quality sequence was trimmed from the Illumina fastq files using the *Trimmomatic* software ([31](#_ENREF_31)), using default options. Reads were aligned to the *A. gambiae* reference genome (AgamP3 ([32](#_ENREF_32))) with the BWA-MEM aligner ([33](#_ENREF_33)). Freebayes v9.9.2-46 ([34](#_ENREF_34)) was used for SNP identification employing standard filters. Non-synonymous and synonymous SNPs were identified with SnpEff 3.4i ([35](#_ENREF_35)) using the Agam3 ([32](#_ENREF_32)) reference. Codon-usage-ratio changes of variants were calculated with the SnpEff annotations and data from the Codon Usage Database (http://www.kazusa.or.jp/codon/). Filtering was performed using SnpSift 4.0e ([35](#_ENREF_35)).

F_ST_ values were calculated using the Weir and Cockerham estimator implemented in VCFtools 0.1.12b ([36](#_ENREF_36)). A list of 231 known immune genes (Table S2) was compiled from the published literature of *A. gambiae* ([1](#_ENREF_1), [37-40](#_ENREF_37)). We excluded genes with unknown chromosome location (UNKN scaffold), mitochondrial genes and genes located in the centromeric “speciation islands” (Figure 1). To be specific, we excluded the 5Mb region adjacent to the X centromere and 2Mb regions from both 2L and 3L where previous studies reported elevated divergence between populations of *A. gambiae* ([41](#_ENREF_41), [42](#_ENREF_42)). Bootstrap p-values ([43](#_ENREF_43), [44](#_ENREF_44)) were generated by comparing the F_ST_ value of the set of immune genes to values computed for a random sample of 231 genes (out of 12,519 total genes, Table S2) repeated 1,000 times. The weighted F_ST_ values were computed using all the potentially functional (non-synonymous or at least 2-fold change in codon usage ratio) SNPs in this set of genes as opposed to averaging per-SNP or per-gene F_ST_ values.

**Reference**

1. Horton AA*, et al.* (2011) The mitogen-activated protein kinome from *Anopheles gambiae*: identification, phylogeny and functional characterization of the ERK, JNK and p38 MAP kinases. *BMC Genomics* 12:574.

2. Altschul SF, Gish W, Miller W, Myers EW, & Lipman DJ (1990) Basic local alignment search tool. *J Mol Biol* 215(3):403-410.

3. Marchler-Bauer A*, et al.* (2011) CDD: a Conserved Domain Database for the functional annotation of proteins. *Nucleic Acids Res* 39(Database issue):D225-229.

4. Bokoch GM (2005) Regulation of innate immunity by Rho GTPases. *Trends in cell biology* 15(3):163-171.

5. Farez MF*, et al.* (2009) Toll-like receptor 2 and poly(ADP-ribose) polymerase 1 promote central nervous system neuroinflammation in progressive EAE. *Nature immunology* 10(9):958-U944.

6. Lindsay SA & Wasserman SA (2013) Conventional and non-conventional *Drosophila* Toll signaling. *Dev Comp Immunol* 42(1):16-24.

7. Jeffrey KL, Camps M, Rommel C, & Mackay CR (2007) Targeting dual-specificity phosphatases: manipulating MAP kinase signalling and immune responses. *Nature reviews. Drug discovery* 6(5):391-403.

8. Salojin K & Oravecz T (2007) Regulation of innate immunity by MAPK dual-specificity phosphatases: knockout models reveal new tricks of old genes. *Journal of leukocyte biology* 81(4):860-869.

9. Hauck ES*, et al.* (2013) Overexpression of phosphatase and tensin homolog improves fitness and decreases *Plasmodium falciparum* development in *Anopheles stephensi*. *Microbes and infection / Institut Pasteur* 15(12):775-787.

10. Marquez AG*, et al.* (2011) Insulin-like peptides in the mosquito *Anopheles stephensi*: Identification and expression in response to diet and infection with *Plasmodium falciparum*. *Gen Comp Endocrinol* 173(2):303-312.

11. Corby-Harris V*, et al.* (2010) Activation of Akt signaling reduces the prevalence and intensity of malaria parasite infection and lifespan in *Anopheles stephensi* mosquitoes. *PLoS Pathog* 6(7):e1001003.

12. Drexler A*, et al.* (2013) Human IGF1 extends lifespan and enhances resistance to *Plasmodium falciparum* infection in the malaria vector *Anopheles stephensi*. *J Exp Biol* 216(Pt 2):208-217.

13. Lim J, Gowda DC, Krishnegowda G, & Luckhart S (2005) Induction of nitric oxide synthase in *Anopheles stephensi* by *Plasmodium falciparum*: mechanism of signaling and the role of parasite glycosylphosphatidylinositols. *Infect Immun* 73(5):2778-2789.

14. Luckhart S*, et al.* (2013) Sustained activation of Akt elicits mitochondrial dysfunction to block *Plasmodium falciparum* infection in the mosquito host. *PLoS Pathog* 9(2):e1003180.

15. Surachetpong W, Pakpour N, Cheung KW, & Luckhart S (2011) Reactive oxygen species-dependent cell signaling regulates the mosquito immune response to *Plasmodium falciparum*. *Antioxidants & redox signaling* 14(6):943-955.

16. Tang D, Kang R, Coyne CB, Zeh HJ, & Lotze MT (2012) PAMPs and DAMPs: signal 0s that spur autophagy and immunity. *Immunological reviews* 249(1):158-175.

17. Crampton A & Luckhart S (2001) The role of As60A, a TGF-beta homolog, in *Anopheles stephensi* innate immunity and defense against *Plasmodium* infection. *Infect Genet Evol* 1(2):131-141.

18. Crampton AL & Luckhart S (2001) Isolation and characterization of As60A, a transforming growth factor-beta gene, from the malaria vector *Anopheles stephensi*. *Cytokine* 13(2):65-74.

19. Lieber MJ & Luckhart S (2004) Transforming growth factor-betas and related gene products in mosquito vectors of human malaria parasites: signaling architecture for immunological crosstalk. *Molecular immunology* 41(10):965-977.

20. Luckhart S*, et al.* (2003) Mammalian transforming growth factor beta1 activated after ingestion by *Anopheles stephensi* modulates mosquito immunity. *Infect Immun* 71(6):3000-3009.

21. Luckhart S, Lieber MJ, Singh N, Zamora R, & Vodovotz Y (2008) Low levels of mammalian TGF-beta1 are protective against malaria parasite infection, a paradox clarified in the mosquito host. *Experimental parasitology* 118(2):290-296.

22. Price I*, et al.* (2013) In vivo, in vitro, and in silico studies suggest a conserved immune module that regulates malaria parasite transmission from mammals to mosquitoes. *J Theor Biol* 334:173-186.

23. Surachetpong W, Singh N, Cheung KW, & Luckhart S (2009) MAPK ERK signaling regulates the TGF-beta1-dependent mosquito response to *Plasmodium falciparum*. *PLoS Pathog* 5(4):e1000366.

24. Vodovotz Y, Zamora R, Lieber MJ, & Luckhart S (2004) Cross-talk between nitric oxide and transforming growth factor-beta1 in malaria. *Current molecular medicine* 4(7):787-797.

25. Stec WJ & Zeidler MP (2011) Drosophila SOCS Proteins. *Journal of signal transduction* 2011:894510.

26. Xu S*, et al.* (1995) MEKK1 phosphorylates MEK1 and MEK2 but does not cause activation of mitogen-activated protein kinase. *Proc Natl Acad Sci U S A* 92(15):6808-6812.

27. Brown J, Wang H, Suttles J, Graves DT, & Martin M (2011) Mammalian target of rapamycin complex 2 (mTORC2) negatively regulates Toll-like receptor 4-mediated inflammatory response via FoxO1. *J Biol Chem* 286(52):44295-44305.

28. McLaughlin MM*, et al.* (1996) Identification of mitogen-activated protein (MAP) kinase-activated protein kinase-3, a novel substrate of CSBP p38 MAP kinase. *J Biol Chem* 271(14):8488-8492.

29. Cargnello M & Roux PP (2011) Activation and function of the MAPKs and their substrates, the MAPK-activated protein kinases. *Microbiology and molecular biology reviews : MMBR* 75(1):50-83.

30. Norris LC*, et al.* (2015) Adaptive introgression in an African malaria mosquito coincident with the increased usage of insecticide-treated bed nets. *Proc Natl Acad Sci U S A* 112(3):815-820.

31. Bolger AM, Lohse M, & Usadel B (2014) Trimmomatic: a flexible trimmer for Illumina sequence data. *Bioinformatics*.

32. Giraldo-Calderon GI*, et al.* (2015) VectorBase: an updated bioinformatics resource for invertebrate vectors and other organisms related with human diseases. *Nucleic Acids Res* 43(Database issue):D707-713.

33. Li H (2013) *Aligning sequence reads, clone sequences and assembly contigs with BWA-MEM* (Cornell University Library).

34. Li H*, et al.* (2009) The Sequence Alignment/Map format and SAMtools. *Bioinformatics* 25(16):2078-2079.

35. Cingolani P*, et al.* (2012) Using *Drosophila melanogaster* as a model for genotoxic chemical mutational studies with a new program, SnpSift. *Frontiers in genetics* 3:35.

36. Danecek P*, et al.* (2011) The variant call format and VCFtools. *Bioinformatics* 27(15):2156-2158.

37. Christophides GK*, et al.* (2002) Immunity-related genes and gene families in *Anopheles gambiae*. *Science* 298(5591):159-165.

38. Cirimotich CM*, et al.* (2011) Natural microbe-mediated refractoriness to *Plasmodium* infection in *Anopheles gambiae*. *Science* 332(6031):855-858.

39. Clayton AM, Dong Y, & Dimopoulos G (2014) The *Anopheles* innate immune system in the defense against malaria infection. *Journal of innate immunity* 6(2):169-181.

40. Stathopoulos S, Neafsey DE, Lawniczak MK, Muskavitch MA, & Christophides GK (2014) Genetic dissection of *Anopheles gambiae* gut epithelial responses to *Serratia marcescens*. *PLoS Pathog* 10(3):e1003897.

41. Turner TL, Hahn MW, & Nuzhdin SV (2005) Genomic islands of speciation in *Anopheles gambiae*. *PLoS Biol* 3(9):e285.

42. White BJ, Cheng C, Simard F, Costantini C, & Besansky NJ (2010) Genetic association of physically unlinked islands of genomic divergence in incipient species of *Anopheles gambiae*. *Mol Ecol* 19(5):925-939.

43. Davison AC & Hinkley DV (1997) *Bootstrap methods and their application* (Cambridge University Press, Cambridge ; New York, NY, USA) pp x, 582 p.

44. North BV, Curtis D, & Sham PC (2002) A note on the calculation of empirical P values from Monte Carlo procedures. *Am J Hum Genet* 71(2):439-441.
